# Dry soils can intensify mesoscale convective systems

**DOI:** 10.1073/pnas.2007998117

**Published:** 2020-08-17

**Authors:** Cornelia Klein, Christopher M. Taylor

**Affiliations:** ^a^UK Centre for Ecology and Hydrology, Wallingford OX10 8BB, United Kingdom;; ^b^Department of Atmospheric and Cryospheric Sciences, University of Innsbruck, 6020 Innsbruck, Austria;; ^c^National Centre for Earth Observation, Wallingford OX10 8BB, United Kingdom

**Keywords:** mesoscale convective systems, propagating convection, soil moisture, dry line

## Abstract

Soil moisture can feed back on rainfall through the impact of surface fluxes on the environment in which convection develops. The vast majority of previous research has focused on the initiation of convection, but in many regions of the world, the majority of rain comes from remotely triggered mesoscale convective systems (MCSs). Here we conduct a systematic observational analysis of soil moisture feedbacks on propagating MCSs anywhere in the world and show a strong positive impact of drier soils on convection within mature MCSs. From thousands of storms captured in satellite imagery over the Sahel, we find that convective cores within MCSs are favored on the downstream side of dry patches ≥200 km across. The effect is particularly strong during the afternoon–evening transition when convection reaches its diurnal peak in intensity and frequency, with dry soils accounting for an additional one in five convective cores. Dry soil patterns intensify MCSs through a combination of convergence, increased instability, and wind shear, all factors that strengthen organized convection. These favorable conditions tend to occur in the vicinity of a surface-induced anomalous displacement of the Sahelian dry line/intertropical discontinuity, suggesting a strong link between dry line dynamics and soil moisture state. Our results have important implications for nowcasting of severe weather in the Sahel and potentially in other MCS hotspot regions of the world.

## Significance

Soil moisture plays a key role in the climate system by affecting rainfall and drought over land. Through its impact on temperature, humidity, and wind in the lower atmosphere, it can influence where thunderstorms initiate. However, in many regions of the world, traveling storm clusters known as mesoscale convective systems (MCSs) are the dominant source of rainfall, and very little is known about their response to surface conditions once triggered. We use satellite observations to demonstrate that dry soils at scales ≥200 km frequently create atmospheric conditions that intensify mature MCSs in the Sahel, long after their initiation. This surface-driven predictability of hazardous weather has potentially important applications, particularly in Africa, where the population is increasingly exposed to flood risk.

In certain regions of the world, soil moisture has an important influence on rainfall (1), with the potential to improve atmospheric predictability (2, 3). When soils dry, evapotranspiration can become water limited (4), and sensible heat warms and deepens the planetary boundary layer (PBL). There have been many studies exploring how soil moisture-induced changes in PBL profiles affect the likelihood of convection (5, 6). At the same time, soil moisture exhibits strong horizontal variability on all scales, due to patterns in antecedent rainfall, soil and vegetation properties, groundwater, irrigation, and wetlands, among other factors. Sensible heat flux gradients on scales ∼10 km upward can drive daytime circulations, which may favor convective initiation (7). There is now good observational evidence globally that such soil moisture heterogeneity is a key factor for understanding where afternoon rain develops (8, 9). These studies are consistent with idealized numerical models, which depict favored zones for convective initiation over drier soils (10, 11). Observational estimates from the Sahel showed this effect to account for one in eight mesoscale convective system (MCS) initiations (12).

By contrast, there has been relatively little work on the impact of soil moisture on remotely triggered propagating convection, yet this is a major contributor to seasonal rainfall in many regions. Implicit in this bias is an assumption that once systems mature and develop their own circulations, they become less sensitive to land surface properties. Individual case studies have suggested that mature MCSs may locally intensify over surfaces with higher evaporation (13, 14). At scales ∼10 km, Taylor and Lebel (15) identified persistent convective scale rainfall patterns in the Sahel from storm to storm. Enhanced sensitivity of convection at these small scales is consistent with the simulated response of convection within an idealized MCS to prestorm PBL moisture perturbations (16). On the other hand, MCSs show a consistent weakening over the wetlands of Lake Chad (∼100 × 200 km) in observations (17) and a numerical model (18).

The MCS studies mentioned above are all from the Sahel, an extensive and largely flat region bordering the Sahara. It provides a “natural laboratory” for studying soil moisture–MCS interactions and is the focus of this systematic analysis of soil moisture controls on convection within mature MCSs anywhere in the world. During boreal summer, it is a global hotspot in intense MCS activity (19), with typical storm return times ∼3 d. During this single rainy season the vegetation develops rapidly, but remains sparse. Evaporation directly from the soil surface is therefore an important component of the surface energy balance in the 1 to 2 d after rainfall (20, 21). This combination produces a daily changing pattern of surface fluxes driven by recent rainfall, on scales ranging from ∼10 km due to an isolated convective cell up to the swath of a long-lived MCS (typically several hundred kilometers north to south by 1,000 km east to west). Where the surface has been recently wetted, the PBL remains cool and shallow (22), and convective instability is weak (23).

Similar to other regions in the United States, South America, Australia, or China, Sahelian MCS initiation preferentially occurs during the afternoon along strong baroclinic gradients, under synoptic conditions characterized by a warm and moist PBL, boundary-layer convergence, and strong vertical wind shear (24). Shear is provided by the low-level southwesterly monsoon flow and the midlevel African easterly jet (AEJ), which develops in response to the regional meridional temperature gradient. Up to 60% of Sahelian MCS initiations have been linked to African easterly waves (AEWs), although it is mesoscale features that ultimately determine storm development (25). Once triggered, MCSs tend to travel westward with the AEJ, weakening and often dissipating in the morning, although some systems can be active for several days. Soil moisture could potentially influence mature MCSs via shear, PBL instability, or convergence. In transition regions such as the Sahel, dry lines provide convergence and have been shown to be sensitive to soil moisture patterns (26). The Sahelian dry line, or intertropical discontinuity (ITD), separates dry Saharan and moist monsoon air masses and favors generation and maintenance of MCSs (27).

## Prestorm Surface and Atmospheric Conditions

Here we use satellite cloud-top temperature observations to analyze convective cores within MCSs ≥15,000 ${km}^{2}$ (*Materials and Methods* and *SI Appendix*, Fig. S1). In particular, we assess preferred core locations relative to prestorm soil moisture structures and interpret the observations using atmospheric reanalysis (ERA5). We first consider the mean surface and atmospheric conditions ahead of storms, based on compositing fields relative to 2,668 cores observed at 1700 Coordinated Universal Time (UTC) (same as local time). We choose that time as it coincides approximately with the diurnal peak in core frequency, maximum vertical extension of MCSs (28), and the expected optimal time for the land to affect convection. In the composite, we have removed cases where the MCS in which the cores are embedded was initiated within 300 km of the core location. This removes effects of soil moisture on convective initiation (12), allowing us to consider feedbacks on remotely triggered systems. We also exclude cases where ERA5 depicts significant midday convection—this step ensures that composited atmospheric features are consistent with prestorm conditions and do not contain artifacts from simulated storms which did not occur.

**Fig. 1.**
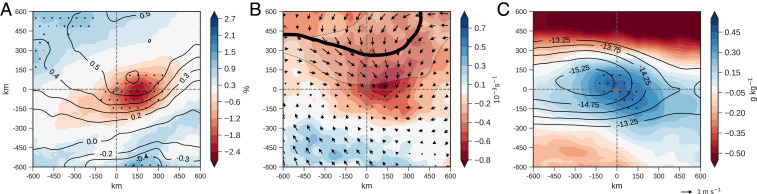
Surface and atmospheric conditions preceding MCSs. (*A–C*) Composites centered on 1700 UTC convective cores within MCSs (gray dot) of (*A*) daytime soil-moisture anomaly (shading, percentage of volumetric soil moisture) and 925-hPa temperature anomaly (contours, K); (*B*) 925-hPa divergence (shading, $10^{-3}s^{-1}$), wind anomaly vectors (m$\cdot s^{-1}$), and 0-line of meridional wind component (black line) representing the ITD dry line with 25% probability range of location (gray shading); and (*C*) 925-hPa specific humidity anomaly (shading, g$\cdot {kg}^{-1}$) and 650- to 925-hPa zonal wind shear (contours, m$\cdot s^{-1}$). Atmospheric variables are for 1200 UTC. Stippling indicates significant anomalies with *P* ≤ 0.01 based on a Welch’s *t* test (*n* = 2,668) relative to the climatology.

Fig. 1*A* shows a clear preference for late afternoon cores to occur in a region of dry soils (*P* ≤ 0.01) extending ∼400 km zonally, with maximum soil dryness approximately 150 km to the east of the core. The pattern is consistent with independent land surface temperature observations (*SI Appendix*, Fig. S2*B*). In the midday reanalysis, there is a mean warm PBL anomaly ∼0.5 K over, and to the north of, the dry soil. Anomalous convergence into the warm PBL anomaly is evident (Fig. 1*B*), accompanied by a southward excursion of the ITD. Additionally (Fig. 1*C*), there is a preference for cores to develop at the center of moist PBL anomalies (∼0.5 g$\cdot {kg}^{-1}$) and in a region of enhanced zonal wind shear. The latter is associated with a locally accelerated AEJ (∼0.9 m$\cdot s^{-1}$) and, to the west of the dry soil, stronger PBL westerlies. As discussed previously, shear, PBL humidity, and the proximity of convergence in the ITD all favor intense organized convection.

**Fig. 2.**
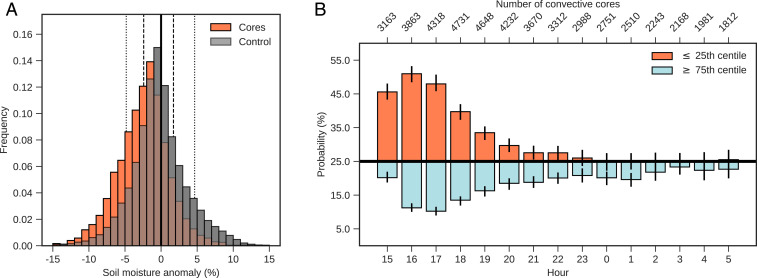
Fraction of convective cores affected by soil moisture conditions. (*A*) Frequency distribution (bars) of soil moisture anomalies (%) at storm time 1700 UTC sampled 150 km upstream of convective core location (orange), for a control sample across the region (gray), and their overlap (brown). Orange (gray) indicates enhanced (suppressed) core probability. Dotted (dashed) vertical lines depict the first and the last decile (quartile). (*B*) Probability for soil moisture anomalies associated with convective cores ≤ the 25th centile (orange) and ≥ the 75th centile of the respective hourly control distribution for 1500 to 0500 UTC. Black horizontal line indicates the 25% expected baseline, and vertical lines denote the 95% confidence interval of a bootstrap with 1,000 samples.

We now consider the frequency distribution of upstream soil moisture anomalies (SMAs) associated with convective cores (Fig. 2*A*). At 1700 UTC, 48% of cores are observed downstream of SMA values less than −2.41%, corresponding to the driest quartile in the climatology. For the soils within the first (driest) decile, the frequency of downstream convective cores is increased by a remarkable 134% compared to climatology. Indeed, a consistent signal of enhanced (suppressed) core frequency downstream of dry (wet) soil is observed from midafternoon to the following morning (Fig. 2*B*), although after 2200 UTC, only the suppression signal associated with wet soils is visible. The impact of SMA is maximized between 1500 and 1800 UTC, when 46% of all cores are observed downstream of soils within their driest quartiles. That enhancement of 21% implies that the soil moisture effect accounts for 1 in 4.8 of all cores in late afternoon.

**Fig. 3.**
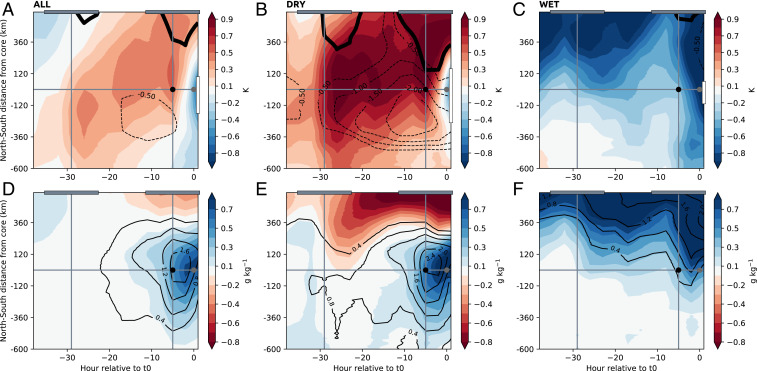
Evolution of the atmosphere until storm time over dry and wet soils. (*A–F*) Composite atmospheric fields in the 36 h ahead of the observed convective cores at 1700 UTC (t0), as a function of north–south distance, and zonally averaged 0 to 200 km upstream of cores, for (*A* and *D*) all convective cores, (*B* and *E*) convective cores over dry soils, and (*C* and *F*) convective cores over wet soils. *A–C* show anomalies of 925-hPa temperature (shading, K), 650- to 925-hPa zonal wind shear (contours, m$\cdot s^{-1}$), and the ITD represented by the 0 line of 925-hPa meridional wind (thick black line). *D–F* depict anomalies of 925-hPa specific humidity (shading, g$\cdot {kg}^{-1}$) and 925-hPa equivalent potential temperature $\Theta_{e}$ (contours, K). Black dot indicates 1200 UTC (sampling time in Fig. 1) and gray dot marks storm time. White bar in *A–C* denotes the average meridional extent of the −1.5% (ALL and DRY case) and 0.1% SMA contour and gray bars highlight daytime (0600 to 1800 UTC).

We now examine how the dry soil patch may prime the atmosphere for intense convection. Fig. 3 shows the mean evolution of atmospheric fields in the 36 h ahead of observed cores. We produce composites based on all cores (Fig. 3 *A* and *D*, “ALL”) and subsets (see *Materials and Methods*) where upstream SMA to the east is negative (Fig. 3 *B* and *E*, “DRY”) or positive (Fig. 3 *C* and *F*, “WET”). The warm PBL anomaly apparent in Fig. 1*A* develops during the previous daytime in ALL, when SMA is already negative (Fig. 3*A*), and is strongly reinforced during the day of the core. The warming is particularly strong in DRY and is responsible for the development of increased convective instability compared to WET, as measured by both convective available potential energy (CAPE) and low-level equivalent potential temperature $\Theta_{e}$ (*SI Appendix*, Fig. S5).

The strong sensitivity to soil moisture evident in Fig. 3, along with the diurnal cycle of the warming, illustrates the central role of sensible heat flux in creating the anomalously warm PBL before MCS arrival. The warming strongly enhances the meridional temperature gradient in DRY, which in turn accelerates the AEJ and hence increases zonal wind shear (Fig. 3*B*). Enhanced heating in DRY also favors anomalous daytime southward migration of the ITD. Considering the development of the humidity anomaly in Fig. 1*C*, we note that the PBL starts to moisten overnight in all three composites. This timing suggests an initial dominant role of moisture advection in the southwesterly monsoon flow, which is maximized overnight (29). Nocturnal moisture advection is not obviously linked to soil moisture state. During the following morning, however, ERA5 depicts strong PBL moistening, which is particularly pronounced in ALL and DRY. Convergence of the low-level flow over the dry soil, strengthened by the nearby ITD, provides an efficient mechanism for this moistening, which more than offsets reduced evapotranspiration from the locally dry soil (*SI Appendix*, Fig. S5*G*). Consequently, we find a larger pre-event $\Theta_{e}$ anomaly (2.4 K) in the DRY case compared to WET (∼0.8 K). Over dry soil, the contribution of temperature dominates the increase in the $\Theta_{e}$ anomaly until 4 h ahead of storm time, when the effect from humidity takes over (*SI Appendix*, Fig. S5*A*).

**Fig. 4.**
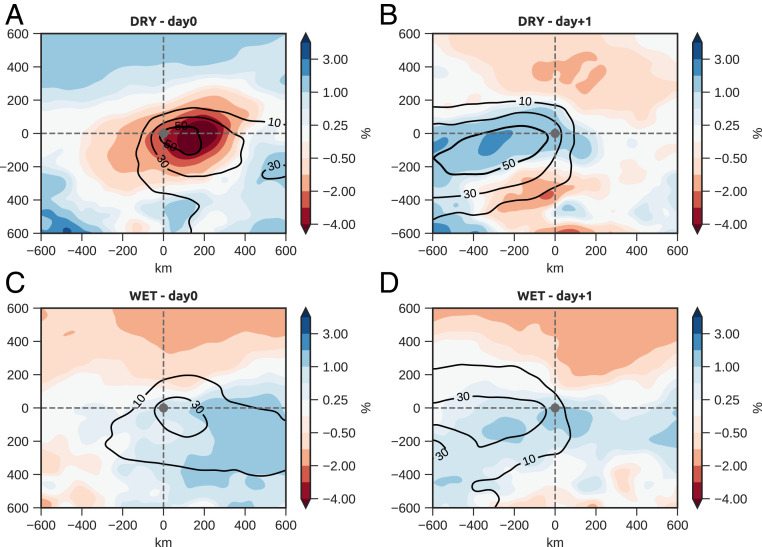
Evolution of soil moisture anomalies and associated rainfall. (*A–D*) Soil moisture anomalies at 1330 UTC (shading, %) on storm day (*A* and *C*) and the following day (*B* and *D*). Contours indicate probabilities (%) for accumulated rainfall ≥5 mm within (*A* and *C*) 1800 UTC (day 1) and 1800 UTC (day 0) and (*B* and *D*) 1800 UTC (day 0) and 1800 UTC (day +1). *A* and *B* (*C* and *D*) show data from the DRY (WET) soil composite.

Not only does dry soil enhance the frequency of convective cores, but also those cores are more intense, as measured by cloud-top temperature (30). On average, cores in DRY are 2.4 °C cooler than in WET (*SI Appendix*, Fig. S3). The most intense cores (colder than −80 °C) make up 25% of the DRY sample, compared to only 9% for WET. The MCSs observed at 1700 UTC in the DRY sample are also on average 39% bigger than their WET-case counterparts, presumably due to a positive effect on convective organization from higher wind shear. The invigorated early evening MCSs in DRY go on to produce more extensive rainfall in subsequent hours (Fig. 4 *B* and *D*). The enhanced overnight rainfall signal in DRY is clearly evident 600 km downstream of the composite location and creates a notably wetter surface to the west.

## Discussion

**Fig. 5.**
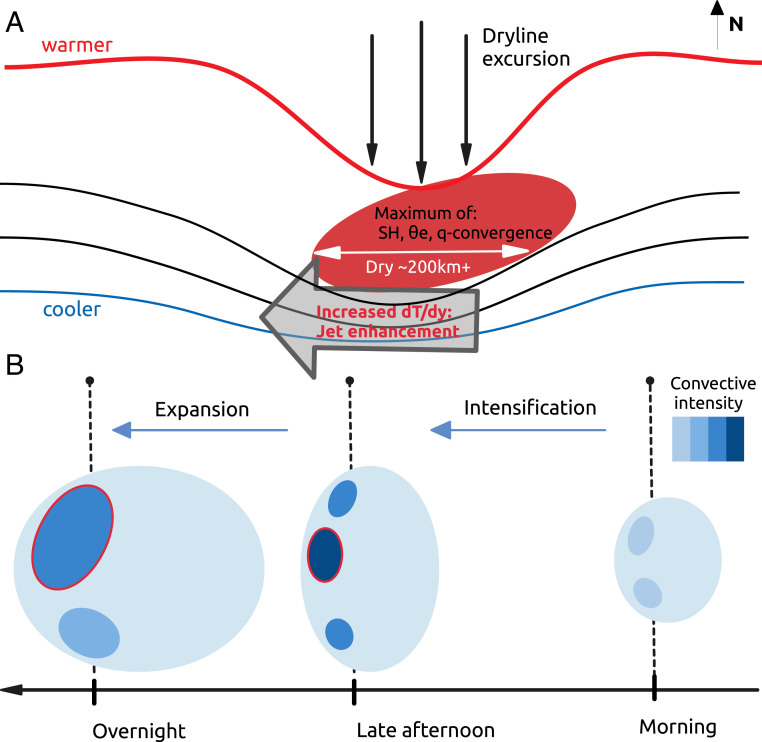
Schematic of dry soil feedback affecting MCS intensity. (*A*) The dry soil area (red ellipse) indicates maximum sensible heat flux (SH), PBL equivalent potential temperature ($\Theta_{e}$), and low-level moisture (q) convergence. Isotherms (lines) promote a southern movement of the ITD (dryline excursion, black arrows), while the strengthened meridional temperature gradient dT/dy enhances the African easterly jet (gray arrow). (*B*) Typical evolution of convective cores (dark ellipses) embedded within a westward propagating MCS over the diurnal cycle (*Right* to *Left*). Convective intensification during late afternoon is particularly marked for cores passing over the dry patch (core circled red), favoring stronger nocturnal cores.

We interpret the preference of convective cores to the west of dry soil as a response via several atmospheric feedback mechanisms (Fig. 5*A*). The dry soil enhances sensible heat at the expense of latent heat, warming the overlying PBL. This daytime forcing on the meridional temperature gradient acts to focus specific features of the monsoon that favor intense MCSs. When the zonal length scale of the dry anomaly is large enough, enhancement of the climatological temperature gradient accelerates the AEJ via the thermal wind and creates a local shear anomaly. In the absence of rain, the enhanced temperature gradient can build over consecutive days, with the AEJ remaining strong overnight. In addition, dry soil provides the mechanism for enhanced heating, which is known to draw the ITD anomalously south during daytime (27). The convergence of the moist monsoon and dry Saharan air masses provides a large-scale source of moisture and supplements more local-scale convergence associated with soil moisture heterogeneity on scales ∼10 km (12). The moisture convergence, coupled with strong PBL temperatures, more than offsets a reduction in local surface evaporation in generating strong convective instability. The combination of this instability and enhanced shear produces more intense convective cores to the west of the dry soil and favors the growth and longevity of MCSs farther downstream (Fig. 5*B*). More intense MCSs in turn favor the creation of extensive new swaths of wet soil oriented WSW (Fig. 4*B*), thus enhancing zonal soil moisture variability on length scales approximately hundreds of kilometers.

In the absence of further storms to disturb the newly established zonal soil moisture gradient, the stage would then be set for a repeat of the feedback cycle, albeit shifted ∼200 km to the east (according to Fig. 4 *A* and *B*). After several rain-free days, the region east of the original core would be drier and warmer than the region to the west. This sequence, whereby one major event favors a second event to the east, amounts to the land surface organizing and distributing convection on the large scale (31). The extent to which it influences the locations of consecutive MCSs is contingent upon synoptic conditions. For example, AEWs may provide an important external forcing on the system and limit the overall impact of the feedback. We find that cores (in particular, in DRY) are favored in northerly flow at 700 hPa. This AEW phase does not especially facilitate MCS activity (25), but does provide relatively settled ridge conditions in the preceding 24 h, which allows the soil to dry out.

This study demonstrates that soil moisture can indeed substantially influence mature MCSs, which reanalysis data indicate is via a combination of dynamic and thermodynamic drivers. Idealized convection-permitting simulations would be valuable in quantifying the contribution of these processes in more detail. While some of the processes highlighted here may be specific to West Africa, it seems likely that similar effects are operating in other MCS hotspot regions of the world. Within West Africa, it opens the opportunity for short-lead predictability of intense rain using localized near-real-time land information from satellites. Improved forecasts would increase preparedness for flash flooding in the region’s rapidly growing cities. The findings also shed light on the strong intensification of Sahelian MCSs observed in recent decades (32), which has been linked to greenhouse gas forcing via strong Saharan warming (33). That warming favors more intense MCSs by enhancing the meridional temperature gradient. Soil moisture feedbacks on length scales approximately hundreds of kilometers may amplify that external forcing. Similar land feedbacks could amplify storms associated with a moister, future atmosphere, which is already more prone to explosive convection, and contribute to strong increases in intense rainfall and dry spells (34). Thus, soil moisture feedback may provide a mechanism that translates global warming into a more extreme Sahelian hydroclimate.

## Materials and Methods

This study is based on satellite observations of MCSs and their relationships with the underlying soil moisture. Our primary data source is thermal infrared imagery from the Spinning Enhanced Visible and Infrared Imager (SEVIRI), which is available from the Meteosat Second Generation (MSG) (35) of satellites at a spatial resolution of 3 km every 15 min. We use data for the Sahel rainy season (June to September) for the years 2006 to 2010. Channel 9 of SEVIRI detects radiation in a band centered at 10.8 μm, and we convert level-1.5 data from the measured counts to brightness temperature using coefficients provided by Eumetsat.

We use these images to analyze convective cores for a Sahelian domain 10 °W to 10 °E, 10 to 20 °N (*SI Appendix*, Fig. S1) within an extensive cold (≤−40 °C, ≥15,000 km^−2^) cloud for every full hour. The cores are associated with intense rain and, unlike the surrounding stratiform cloud shield of the MCS, are directly connected to the PBL, via deep updrafts. To identify cores, we apply a two-dimensional wavelet scale decomposition to the temperature field, following our previous work (30). Center points of convective cores are identified from significant local power maxima ($\geq \sqrt{scale}$) within a two-dimensional footprint of 30 × 30 ${km}^{2}$ and are assigned the scale of the absolute power maximum across the scale dimension. We consider power maxima only for scales 9 to 65 km for our analyses. Within this scale range, peak rain rates are centered on cores, of which 84% are associated with convective rainfall (30). Indeed, spaceborne precipitation radar shows that ∼41% of cores are associated with intense rainfall rates exceeding ≥30 mm$\cdot h^{-1}$. For all analyses, we consider only cores close to the MCS edge by filtering out cases where a cold cloud (≤−40 °C) was present 2 h or more ahead of storm time. We seek to minimize orographic effects by excluding cores that occur over topography ≥450 m; at 1700 UTC this amounts to 13% of the dataset. Finally, we exclude cores embedded within MCSs that were initiated within 300 km of core location (33%) by backtracking overlapping cold cloud (12) to obtain a sample of mature, propagating MCSs. The above filtering steps result in 4,318 convective cores at 1700 UTC.

We wish to characterize transient soil moisture features which are known to drive daytime PBL variability (22). For this, we rely primarily on passive microwave-based surface soil moisture estimates. We use data from the Advanced Microwave Scanning Radiometer–Earth Observing System (AMSR-E) (36) on board the polar-orbiting Aqua satellite, specifically the level-3 0.25° gridded product from the Land Parameter Retrieval Model (37). This provides snapshots of Sahelian volumetric soil moisture typically once per day, either around 0130 or 1330 local time. For each pixel and time of day, we create anomalies relative to a long-term (2002 to 2011) climatology based on an 11-d moving window. We calculate the enhancement of core frequency depending on upstream soil moisture conditions in Fig. 2 with respect to a climatology of soil moisture anomalies sampled surrounding each core. Per core, 26 points are sampled at ±150-km intervals up to ±600 km east–west distance and at 0 km and ±150 km north–south distance.

We analyze independent observations of clear-sky land surface temperature (LST) (38) to confirm that SMAs are indeed associated with anomalous surface fluxes. Following Taylor et al. (12), we use 15-min LST images, also produced from MSG data at 3 km resolution (39). We apply additional cloud screening to the standard product (14) and compute LST anomalies from the monthly mean diurnal cycle at each pixel and for each image between 0800 and 1330 UTC. The daily mean LST anomaly (LSTA) is then the average of all available data during that period of the day and can be used as a proxy for soil moisture. *SI Appendix*, Fig. S2 illustrates strong negative correlations between LSTA and SMA and provides independent confirmation of our key finding.

Outside of field campaigns, there are no observations of the atmospheric response to the land surface at spatial and temporal scales suitable for this study. We therefore rely on atmospheric reanalysis, based on assimilation of in situ and satellite observations within a numerical weather prediction model. We use ERA5 (40), the state of the art in reanalysis, which provides hourly output at 0.25° resolution. Importantly, unlike its predecessors, ERA5 assimilates satellite soil moisture from active microwave. This provides additional spatial information to constrain surface fluxes, which should improve the depiction of the daytime PBL. *SI Appendix*, Fig. S4 shows there is indeed broad consistency between the composite SMA patterns derived from our passive microwave dataset and ERA5 and its assimilation of independent active microwave data. We expect that averaged over many days, ERA5 will produce a reasonable characterization of the large-scale structure of the atmosphere, but will not produce realistic convective systems, as storm occurrence and location are not well constrained by observations. We therefore exclude cases with preexisting convection in the reanalysis, as defined by midday 500-hPa vertical velocity ≤ −0.4 Pa$\cdot s^{-1}$ within a 200 × 200-km box upstream of the afternoon core location (*n* = 2,668, 1700 UTC). With this filtering, we find that our composite results (e.g., Fig. 1) show a physically realistic picture of the pre-MCS environment. In particular, the presence of warm PBL anomalies over dry soils, and shear and humidity maxima in locations where cores are subsequently observed, provides strong support for our use of ERA5 data in interpreting prestorm atmospheric processes.

To evaluate the effect of soil moisture on our analyses, we create additional DRY and WET composites, based on SMA values averaged over a radius of 75 km, centered 150 km upstream of each core. The DRY (WET) sample contains the driest (wettest) 20% of the SMA distribution associated with cores, as shown in Fig. 2*A* for 1700 UTC. Note that we do not use a larger sample because of the strong tendency toward dry soils associated with convective cores; i.e., the “wettest” 25% of the SMA distribution would include negative values. In addition, we constrain the DRY/WET subsamples to conditions for which the climatological 925-hPa specific humidity at core location is ≥14 g$\cdot {kg}^{-1}$ (*n* = 3,752, 1700 UTC). This ensures comparability of climatological atmospheric moisture between the two cases (*n* = 750, 1700 UTC). Finally, we use the 3-hourly CMORPH precipitation dataset (41) at 0.25° to compare precipitation probabilities under WET and DRY soil conditions (Fig. 4).

## Supplementary Material

Supplementary File

## Data Availability

All datasets used in this study are publicly available. We obtained MSG data from http://www.eumetsat.int/, soil moisture from https://disc.gsfc.nasa.gov/, LST from the https://landsaf.ipma.pt./, ERA5 from https://climate.copernicus.eu/climate-reanalysis, and CMORPH from https://www.cpc.ncep.noaa.gov/. All code used in this study to perform the analyses and to create the figures can be made available upon request to the corresponding author.
