# Supplementary material for: Dry soils can intensify mesoscale convective systems
Source: Proc Natl Acad Sci U S A. 2020 Aug 17;117(35):21132–7. doi: 10.1073/pnas.2007998117 (PMC7474668; doi:10.1073/pnas.2007998117)
Supplement: Supplementary File [file pnas.2007998117.sapp.pdf]

Supplementary Information for

Dry soils can intensify mesoscale convective systems

Cornelia Klein, Christopher M Taylor

Corresponding author: Cornelia Klein.
E-mail: cornkle@ceh.ac.uk

This PDF file includes:

Figs. S1 to S5

www.pnas.org/cgi/doi/10.1073/pnas.2007998117

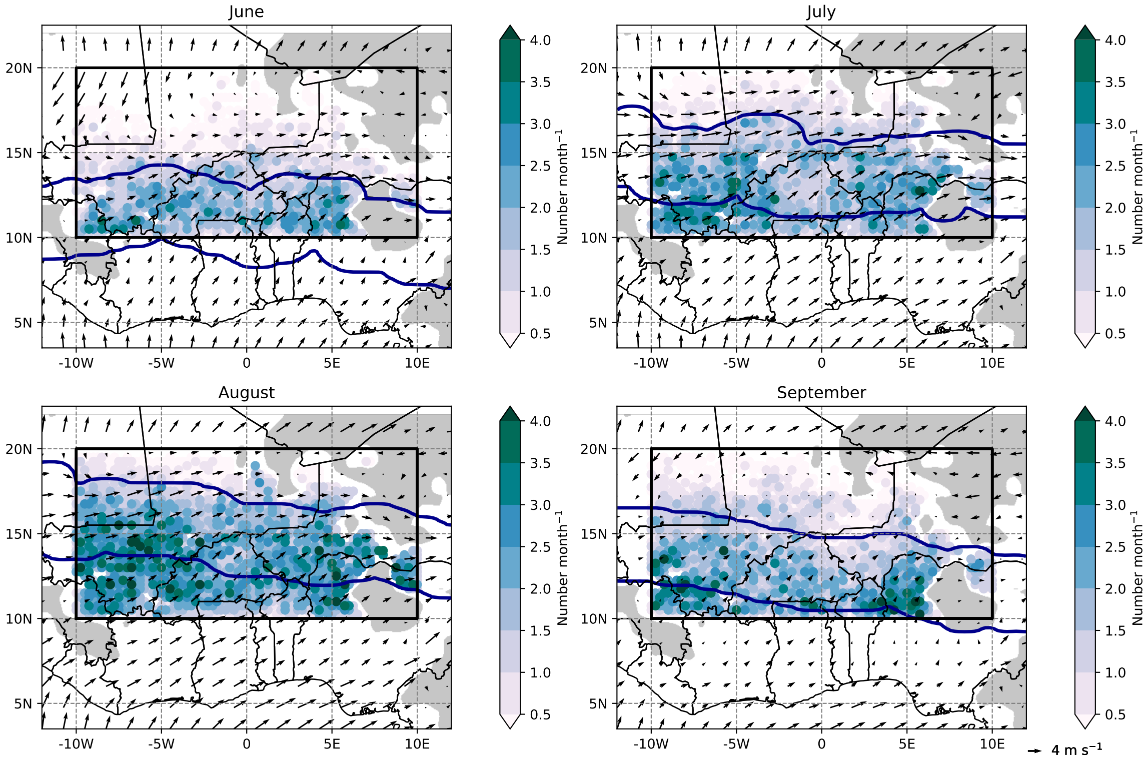

Fig. S1. Maps of convective core locations. Average monthly core frequency for JJAS 2006-2010 aggregated on ERA5-grid (0.25°), 925hPa wind vectors (m s^{-1}) and region of strongest (10th centile) easterly 650hPa zonal wind per north-south slice, indicating the position of the African Easterly Jet (blue contours). Grey shading marks topography $\geq$ 450m.

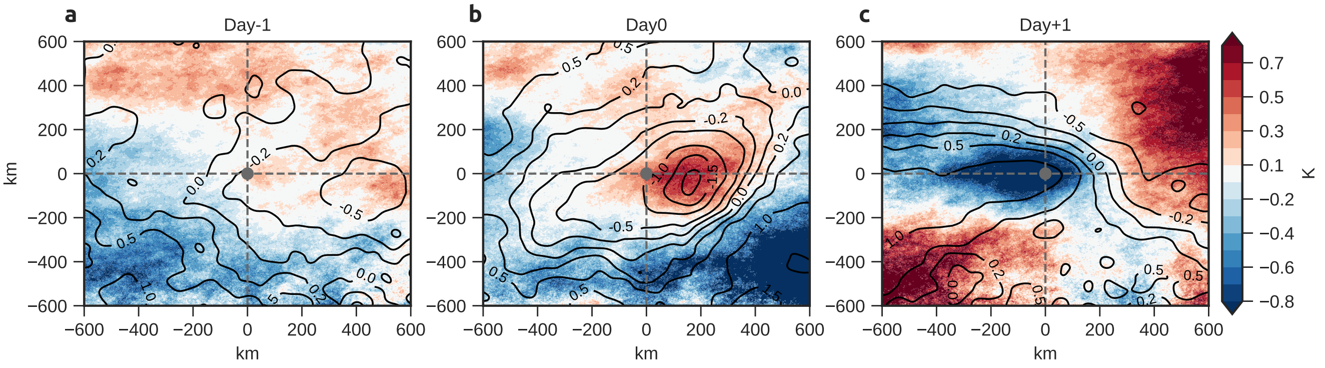

Fig. S2. Comparison of land surface temperature and soil moisture anomalies. Composites centred on 1700UTC convective cores for 06-1300UTC land-surface temperature anomalies (shading, K) and daytime soil moisture anomalies (contours, %) for (a) preceding day (day-1), (b) storm day (day0) and (c) following day (day+1).

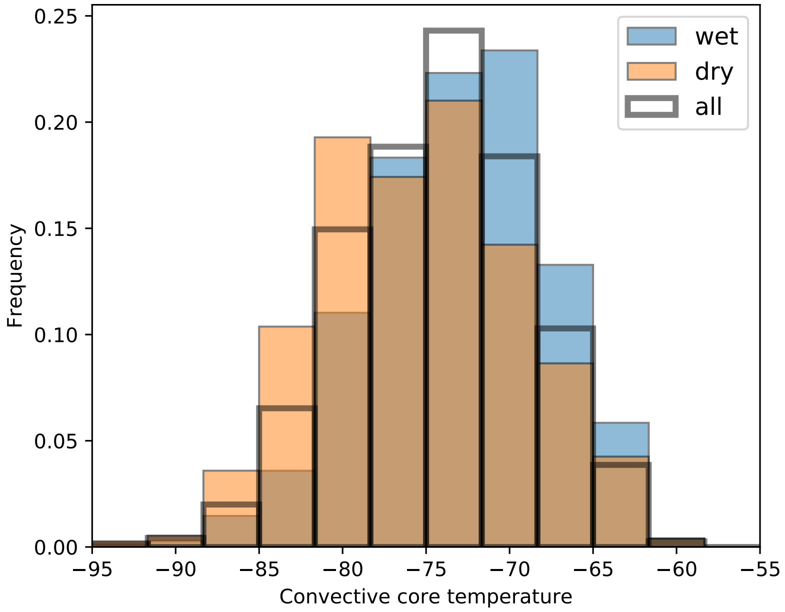

Fig. S3. Distribution of convective core temperatures. Minimum temperatures for convective cores associated with the DRY soil and WET soil cases. Black bars include all soil conditions (n=3752).

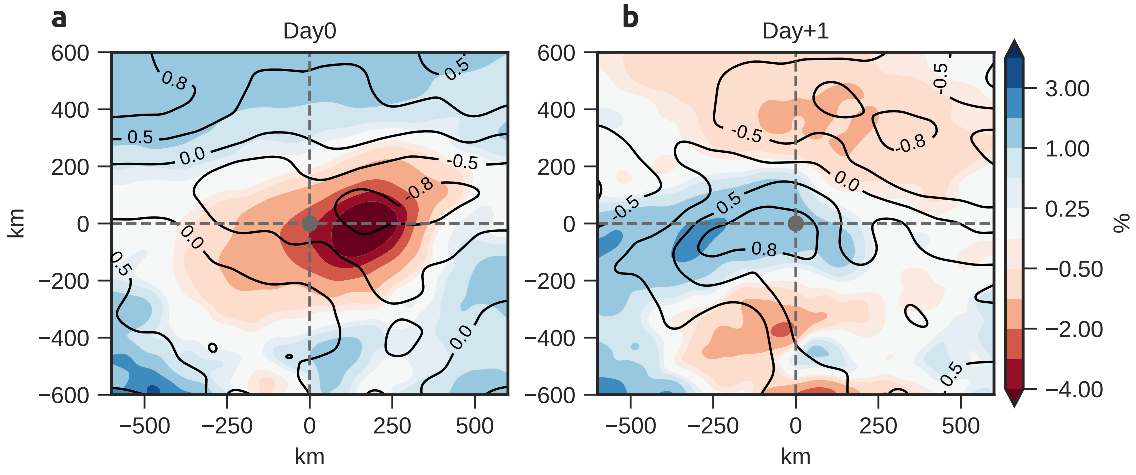

Fig. S4. Comparison of soil moisture anomalies in observations and ERA5. SMA composites centred on 1700UTC convective cores of the DRY case for AMSR-E (shading, %) and anomaly of ERA5 volumetric soil water in layer 1 (contours, %) for (a) storm day (day0) and (b) following day (day+1).

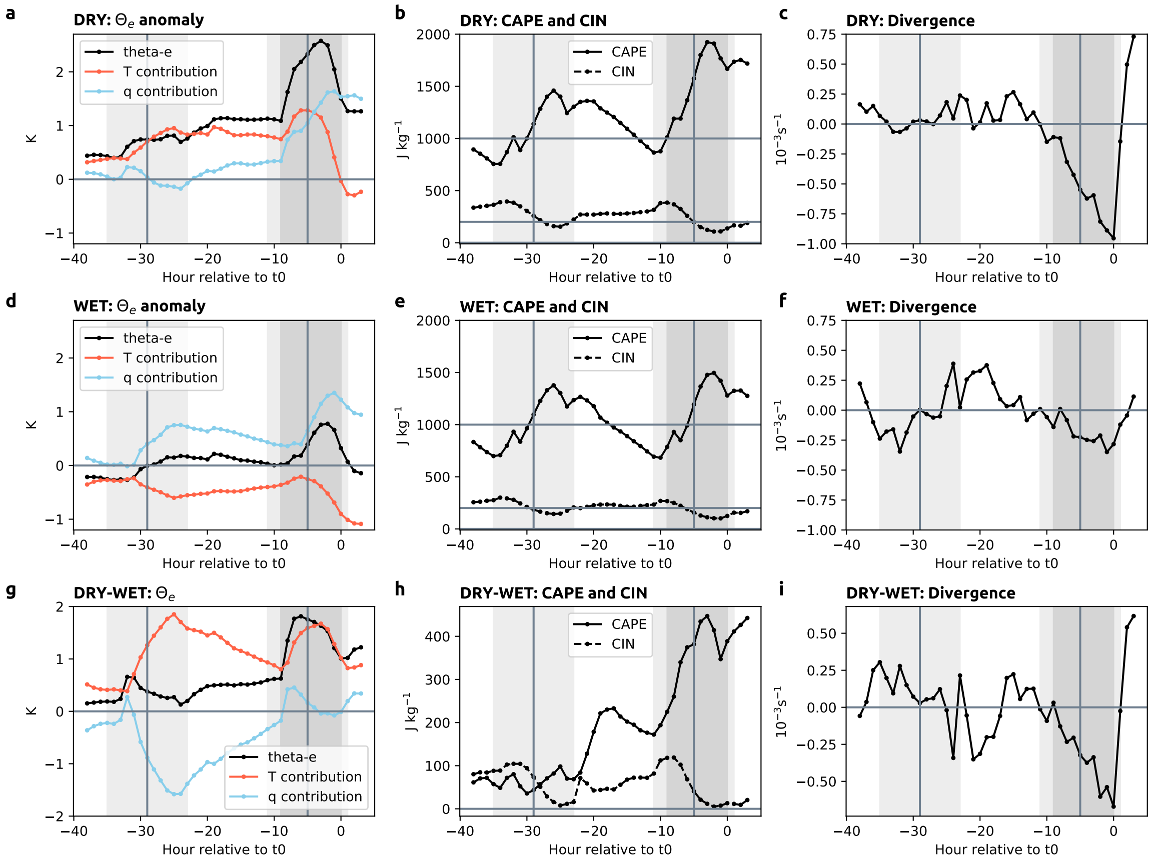

Fig. S5. Local evolution of instability measures. ERA5 composite means for a 50x50km box centred on 1700UTC convective cores showing the evolution of (a,d,g) 925hPa Θ_e and entropy contribution from temperature and specific humidity (K), (b,e,h) total column CAPE and CIN (J kg^{-1}), and (c,f,i) 925hPa divergence (10^{-3}s^{-1}) for 38 hours until storm time. Top and middle rows show Θ_e anomalies, CAPE, CIN, and divergence for the DRY and WET case separately while bottom row shows difference values DRY-WET. Light grey shading indicates daytime hours and dark grey shading highlights the time window (0800-1700UTC) of anomalous daytime temperature increase for DRY on storm day. Vertical lines mark 1200UTC.
